# Determinants of viral load suppression among orphaned and vulnerable children living with HIV on ART in Tanzania

**DOI:** 10.3389/fpubh.2023.1076614

**Published:** 2023-03-17

**Authors:** Amal Ally, Amon Exavery, John Charles, Levina Kikoyo, Remmy Mseya, Asheri Barankena, Rose Fovo, Akwila Temu

**Affiliations:** Pact Tanzania, Dar es Salaam, Tanzania

**Keywords:** viral suppression, retention, adherence, HIV, orphans

## Abstract

**Introduction:**

In Tanzania, only 66% children 0–14 years living with HIV know their HIV status, 66% are on treatment while 47% of children on ART are virally suppressed. Although retention on ART and poor adherence remain a challenge for children living with HIV, orphans and vulnerable children (OVC) face a greater limitation of access to and utilization of comprehensive HIV care and treatment. In response to this, the current study assessed the determinants of viral load suppression (VLS) among OVC aged 0–14 years living with HIV enrolled in HIV interventions.

**Methods:**

This was a cross-sectional study that used secondary data collected by the USAID Kizazi Kipya project in 81 district councils of Tanzania. Included in this study are 1,980 orphans and vulnerable children living with HIV (OVCLHIV) (0–14 years) enrolled and served by the project for 24 months. Data analysis involved multivariable logistic regression, with viral load suppression as the outcome of interest and HIV interventions as the main independent variables.

**Results:**

The overall VLS rate among the OVCLHIV was 85.3%. This rate increased from 85.3, 89.9, 97.6 to 98.8% after 6, 12, 18, and 24 months of retention on ART, respectively. Similar rates were observed as the duration of adherence to ART increased. In the multivariable analysis, OVCLHIV attending people living with HIV (PLHIV) support groups were 411 times more likely to be virally suppressed than those not attending (aOR = 411.25, 95% CI 168.2–1,005.4). OVCLHIV with health insurance were 6 times more likely to achieve viral suppression than those without (aOR = 6.05, 95% CI 3.28–11.15). OVCLHIV with >95% adherence to ART were 149 times more likely to be virally suppressed than those not adherent to ART (aOR = 148.96, 95% CI 42.6–520.6, *p* < 0.001). Other significant factors included food security and family size. OVCLHIV reached by the different HIV community-based interventions were more likely to be virally suppressed than those who were not.

**Discussion:**

To advance viral suppression, efforts should be made to ensure that all OVCLHIV are reached by community-based interventions as well as integrating food support in HIV treatment interventions.

## Background

HIV/AIDS remains the serious public health challenge worldwide especially in low- and middle-income countries. Statistics show that 1.5 million new HIV infections and 680,000 deaths from AIDS-related causes occurred in 2020. There were 37.7 million people living with HIV, including 10.2 million who were not on HIV treatment. Among those not on treatment, about 4.1 million did not know their HIV-positive status and 6.1 million knew their HIV status but could not access treatment (1). Major gaps in the testing of infants and children exposed to HIV have left more than two fifths of the children living with HIV undiagnosed (2). In 2020, the number of children not on treatment globally is estimated to be 800,000 children (aged 0–14 years). Just 40% of children living with HIV had suppressed viral loads, compared to 67% of adults. This treatment gap suggested existence of more HIV treatment challenges for children than adults (2).

East and Southern Africa is the region hardest hit by the HIV epidemic, accounting for ~55% of all people and two thirds of all children living with HIV. About 20.6 million adults and children are living with HIV, 670,000 are newly infected with HIV and 310,000 deaths due to AIDS (1).

Tanzania has one of the youngest populations on the African continent and globally (3). The 2012 Population and Housing Census results show that, Tanzania has a population of 44.9 million of which 44.1% are young population <15 years (4). Among the population aged 0–14 years, 71.6% are under the age of 10 years (5). In 2020, it was estimated that around 1.7 million people were living with HIV in Tanzania, 68,000 people were newly infected with HIV, and 32,000 people died from AIDS- related illness (1).

HIV prevalence among children aged 0–14 years is estimated to be 0.4% (0.3% of males and 0.5% of females). Only 66% children 0–14 years living with HIV know their HIV status, 66% are on treatment while 47% of children on ART are virally suppressed (2). Treatment coverage among children living with HIV remains lower than treatment coverage among adults, and it is far short of the 1.6 million targets set for 2018 (6).

HIV treatment involves taking medicines that suppress HIV replication in the body. All children <15 years of age who have a confirmed diagnosis of HIV, regardless of WHO clinical stage or CD4 cell count, must take ART every day as prescribed by health care provider to reduce the amount of HIV (viral load) in the blood to a very low level, but this is only possible if ART adherence is maintained (7, 8). Children living with HIV who initiate ART upon diagnosis and adhere to their treatment regimen can have near-normal life expectancy (9). Consequences of poor adherence to ART in children include treatment failure, HIV drug resistance, increased morbidity and mortality as well as growth and developmental faltering (2). Extant evidence from the United Nations Programme on HIV/AIDS (UNAIDS), shows that, the overall VLS among children living with HIV (CLHIV) aged 0–14 years, was 47% in 2020 (2). Trials in different countries have demonstrated that community-based intervention improves HIV care services among children living with HIV and AIDS (10). However, in Tanzania there has been limited documentation of the contribution of community-based interventions on improving health outcomes of children living with HIV who are on-ART (11). Therefore, viral suppression in orphans and vulnerable children living with HIV remains a major challenge in Tanzania and hence a need to be addressed.

This study aimed to determine successful community-based interventions in improving viral load suppression in children living with HIV enrolled in OVC interventions in Tanzania.

## Methods

### Data sources

Data for this study was obtained from the community-based USAID- funded Kizazi Kipya project (2016–2021) in Tanzania. The USAID Kizazi Kipya project aimed to increase the uptake of age-appropriate HIV- related and other health services and social services for improved health and wellbeing by OVC, adolescent and their families. HIV community-based data is collected by Community Case Workers (CCWs) from caregivers through self-reports during beneficiary screening and enrollment into project using Family and Child Asset Assessment (FCAA), HIV Risk services and Adherence Assessment (HRAA), HIV Risk services and Adherence Quarterly Monitoring (HRAQM) tool, Improved Community Health Fund (iCHF) received form and National Most Vulnerable Children (MVC) Monthly Service Tracking tool from October 2018 to September 2020. Viral load data for this study was obtained from HIV Care and Treatment Centers (CTC) through PEPFAR care and treatment implementing partners. Through CCWs, retention and adherence to ART was monitored through monthly case management visits at the household level, linked OVCLHIV to PLHIV or age-appropriate clinic, provided disclosure support to 8+ years OVCLHIV, followed-up on OVCLHIV interruption in treatment and missed appointments, provided escorted referrals to OVCLHIV with high viral loads to attend enhanced adherence session, provided iCHF to OVCLHIV households. CCWs are government carders (12) trained on National Integrated Case Management System (NICMS) to provide household level case management services to OVCLHIV and their families.

### Study area

This study used secondary data originating from the USAID Kizazi Kipya project. The data stem from 81 district councils in 25 regions of Tanzania. The regions are Arusha, Dar-es-Salaam, Dodoma, Geita, Iringa, Kagera, Katavi, Kigoma, Kilimanjaro, Mara, Mbeya, Mjini Magharibi, Morogoro, Mtwara, Mwanza, Njombe, Pwani, Rukwa, Ruvuma, Shinyanga, Simiyu, Singida, Songwe, Tabora, and Tanga.

The USAID Kizazi Kipya was a 5-year OVC project (July 2016–June 2021) funded by the President's Emergency Plan for AIDS Relief (PEPFAR) through the United States Agency for International Development (USAID) and implemented by Pact Tanzania. The project goal was to improve health and social wellbeing of OVC, young people and their families through strategic service delivery and support (13, 14). The project also addressed critical barriers to service access, uptake, and adherence in order to scale up high-impact service delivery, advance 95-95-95, and improve health and social wellbeing outcomes among OVC and their families (14).

Supporting the Government of Tanzania to advance the global 95-95-95 goals, USAID Kizazi Kipya project delivered rapid scale-up of proven, family-centered, impact mitigation efforts for OVC, reinforced with cross-sectoral, evidence-driven interventions to reduce HIV incidence while improving performance across the HIV treatment outcome (14). The project used CCWs who are the government cadre of volunteers trained on the National Integrated Case Management System (NICMS) to provide services to OVC and their caregivers (12). Through OVC program, a comprehensive OVCLHIV package of interventions are implemented to children and their families with known high-risk characteristics (children living with HIV, HIV exposed infants, victims of abuse, children living and working in the streets, children in the mining, children of female sex workers etc.) (14).

OVC with high-risk characteristics remains at several risks to realizing their potential, especially limited access to HIV related services, sexual and reproductive health information, unintended pregnancy, and sexually transmitted infections (STIs), particularly HIV infection (14). Due to OVCLHIV vulnerability, HIV intervention package are designed to support OVCLHIV to sustain long-term adherence to treatment and retention in ART care contributing to viral suppression and hence attainment of the third 95% target of UNAIDS (14). OVCLHIV receive tailored-made service package designed to support adherence to ART and retention in ART to OVCLHIV 0–14 years. The service included (i) ART retention and adherence quarterly monitoring by CCWs (ii) disclosure support for OVCLHIV 8+ years (iii) provision of health insurance coverage (iCHF) to OVCLHIV families (iv) linkage of OVCLHIV to PLHIV groups (13, 14).

### Study population

The study population comprised of 1,980 OVCLHIV who were enrolled in the USAID Kizazi Kipya project and reached with HIV intervention for 24 months from October 1st, 2018 to September 30th, 2020. OVCLHIV who were on ART with valid Care and Treatment Clinic (CTC) IDs were enrolled in the study. OVCLHIV included in this study were aged 0–14 years; slightly more than a half of the OVCLHIV were female amounting to 1,016 (51.3%).

### Study design

This was a cross-sectional study in design that used program monitoring data collected once at the end of the study period. OVCLHIV in this study were assessed at the 24th month (in September 2020) following continuous provision of HIV interventions from October 1st, 2018 to September 30th, 2020 to determine Viral Load Suppression (VLS).

### Variables

The outcome variable for this study was OVCLHIV viral load suppression. OVCLHIV who received ART for 6 months were eligible for viral load test. OVCLHIV who had their viral load test and their viral load results were <1,000 copies per milliliter of blood were virally suppressed. Those who had their viral load test and the viral load results were 1,000 or more copies of blood per milliliter of blood were not virally suppressed. This definition is in accordance with the National HIV viral load (HVL) testing and HIV management guidelines (15, 16). Structurally, it was a binary variable, statistically coded as “0” if viral suppression was not achieved, and “1” if the child was virally suppressed.

The main independent variables constituted OVCLHIV community-based interventions (CCWs household monitoring visits, disclosure support, attendance in PLHIV groups or age-appropriate clinic and health insurance coverage). Caregiver demographic, socio-economic and household economic characteristics were included as indirect independent variables to control their potential confounding effect on viral suppression. Adherence to ART was also included as an independent variable which was defined as taking 95% (7, 8) or more of prescribed medication in the past one months. During monthly household visit by CCWs, OVCLHIV ARV adherence was assessed using HIV Risk Assessment Quarterly Monitoring (HRAQM) tool. OVCLHIV ARV treatment was therefore self-reported by caregiver to CCWs. As self- reported by caregiver, CCW records whether OVCLHIV has started ART, frequency the child was supposed to take her/his ART, and number of times missed her/his ART medication during the past 30 days. If a OVCLHIV missed 0–1 and 0–3 doses of ART prescribed once and twice per day, respectively, then OVCLHIV was classified as adherent and was statistically coded “1” and if OVCLHIV missed 2 or more and 4 or more doses of ART prescribed once or twice per day, respectively, then OVCLHIV was classified as not adherent and was coded “0”.

CCWs provide appropriate ART treatment and adherence services during monthly household visits. During household visits, CCWs trained in ART retention and adherence provided support and address barriers on missed clinic appointments, interruption in treatment, provision of escorted referrals (as needed) to health facility and support completion and deliver a tailored—made service package to OVCLHIV. HRAQM tool was used to assess OVCLHIV retention and adherence status by CCWs at household level. If OVCLHIV received monthly monitoring visits by CCWs, was coded as “1” and those who did not received monthly monitoring visits were coded as “0”.

As per the Tanzanian government's guidelines (17), disclosure of the HIV status of a child should be discussed with a caregiver as early as possible by health provider. Disclose process can start as early as 4–6 years of child's age. At about 8+ years, it is recommended that child's full disclosure of HIV and AIDS status should be done in a caring and supportive manner. Through routine case management visits conducted by the CCWs at the household level, CCWs use HRAQM screening tool to establish need for child age- appropriate HIV disclosure support, support OVCLHIV and their caregivers to the health facilities for disclosure counseling and provide close support to caregiver during disclosure process. Disclosure support was done to OVCLHIV when he/she was aged 8 years and above. This was a binary variable that was statistically coded as “1” If the OVCLHIV aged 8 years or more had been informed of his/her own HIV status by his/her caregiver and “0” if not.

Community Case workers conducts household case management visit to assess OVCLHIV attendance to PLHIV support groups or age-appropriate clinics. Through CCWs, OVCLHIV and their caregivers were provided referrals and linkage to attend PLHIV support groups or age-appropriate clinics for continuous psychosocial support. Attendance in PLHIV groups or age appropriate was measured by assessing if OVCLHIV attended PLHIV support group or age-appropriate clinics. This was a binary variable that was statistically coded as “1” If the OVCLHIV was linked to PLHIV support group or age-appropriate clinics and “0” If the OVCLHIV did not attend PLHIV support group or age-appropriate clinics.

OVCLHIV on ART face a myriad of medical, psychological, and social challenges including side effects of ART, higher vulnerability to opportunistic infections, stigma, discrimination and difficulty to access and utilize age appropriate and child friendly health services. Several factors can be attributed to low access of health care services, including the cost of health care services. As part of OVCLHIV service package, CCWs support provision of health insurance cards exclusively to OVCLHIV and their families to address barriers to access health services hindering ART retention and adherence to not only achieve sustained viral load suppression, but to also improve the resilience and overall wellbeing of these children. Health insurance was covered for the whole household. Measuring health insurance coverage was done by assessing if OVCLHIV was provided with health insurance coverage. This was a binary variable that was statistically coded as “1” If the OVCLHIV was provided with health insurance and “0” If the OVCLHIV was not provided with health insurance.

### Data analysis

Data analysis was conducted using Stata statistical software (version 16.0). Exploratory analysis in the form of one-way tabulations (descriptive analysis) were conducted to determine distribution across different variables. This process yielded proportions, averages, and total numbers for each of the key variables.

Through cross-tabulations, a comparison of each of the outcomes, e.g., variation of viral suppression among OVCLHIV of different clinical and social backgrounds was conducted and a Chi-square test (^χ^) was used to assess the degree of association between two variables.

Multivariable analysis studying one outcome (dependent) variable with two or more independent variables at the same time—was conducted only for viral suppression. Since viral suppression was a binary variable, it was modeled using logistic regression (18, 19). Adjusted odds ratios of the predictive effect of the independent variables on viral suppression and their corresponding 95% confidence intervals and *p*-values were presented. All statistical inferences were made at 5% (α = 0.05) level of significance.

## Results

### Profile of OVCLHIV

This study included 1,980 OVCLHIV aged 0–14 years on ART in 81 councils across the country. Of the total OVCLHIV sample, female amounted to 1,016 (51.3%) and male 964 (48.7%). A half (50.1%) of the OVCLHIV were aged between 10 and 14 years, 63.8% (1,264) of the OVCLHIV were in school, 12.8% were not school age (age below 6 years), 13.5% had unknown school status and 10.4% were out of school (Table 1).

**Table 1:** Profile of orphans and vulnerable children living with HIV (OVCLHIV).

**Variable**	**Number of OVCLHIV (*n*)**	**Percent (%)**
Overall	1,980	100.0
**OVCLHIV sex**		
Female	1,016	51.3
Male	964	48.7
**OVCLHIV age**		
00–04 years	323	16.3
05–09 years	666	33.6
10–14 years	991	50.1
**School enrolment status**		
In school	1,264	63.8
Not school aged	244	12.3
Out of school	205	10.4
Unknown	267	13.5
**Caregiver sex**		
Female	1,424	71.9
Male	556	28.1
**Caregiver age**		
18–24 years	15	0.8
25–45 years	886	44.8
46–65 years	832	42.0
66+ years	247	12.5
**Caregiver HIV status**		
Negative	521	26.3
Positive	1,395	70.5
Undisclosed	28	1.4
Unknown	36	1.8
**Household food security**		
Secured	1,616	81.6
Not secured	364	18.4
**Household size**		
2 people	706	35.7
3 people	490	24.8
4 people	370	18.7
5 people	414	20.9
**Place of residence**		
Rural	1,257	63.5
Urban	723	36.5

In relation to OVCLHIV caregiver characteristics, 70.5% (1,395) of the OVCLHIV had HIV positive caregivers, 26.3% (521) had HIV negative caregivers, 1.4% (28) had caregivers with undisclosed HIV status, and 1.8% (36) had caregivers with unknown HIV status. The majority of OVCLHIV (71.9%) had female caregivers; and close to a third (63.5%) resided in rural areas. Overall, OVCLHIV household size ranged between 2 and 5 family members. About a third OVCLHIV (35%) were from households with 2 members. With respect to food security (availability and accessibility of food), the majority of OVCLHIV (81.6%) were in food secure households and only 18.4% of OVCLHIV household were food insecure (Table 1).

### Coverage of OVCLHIV interventions

**Figure 1:**
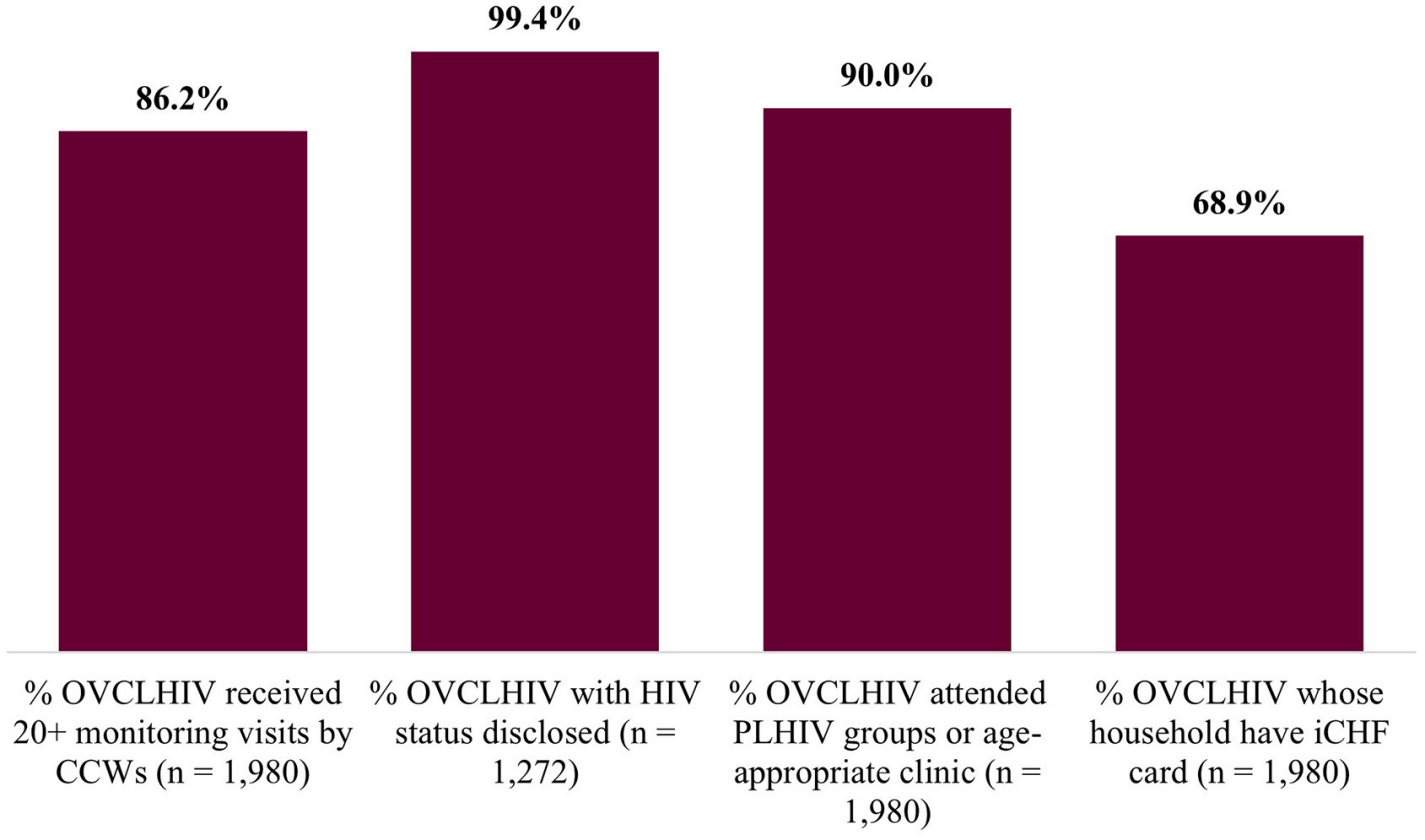
Coverage of interventions.

Figure 1 shows the proportion of OVCLHIV intervention outcomes among the 1,980 OVCLHIV who received an evidence-based package of OVCLHIV services delivered by the CCWs through regular household visits designed to increase OVCLHIV retention and adherence to ART. A majority (86.2%) of OVCLHIV received at least a single visit in 20+ months. Almost all OVCLHIV who were aged 8+ years (99.4%) had their HIV status disclosed, 90.0% of OVCLHIV were supported to attend PLHIV groups and 68.9% of the OVCLHIV's household had received health insurance (iCHF cards).

### OVCLHIV viral suppression by OVCLHIV interventions and other characteristics

This subsection shows viral suppression rates by different intervention received by the OVCLHIV. The rates are also compared across different levels of socio demographic, household, and geographical characteristics (Table 2).

**Table 2:** OVCLHIV viral suppression rate by interventions and other background characteristics (*N* = 1,980).

	***N***	**% Virally suppressed**	***P*–value**
Overall	1,980	85.3	—
Attending PLHIV group			<0.001
No	198	5.6	
Yes	1,782	94.1	
HIV disclosure status (only age 8+ years)			<0.001
Undisclosed	7	0.0	
Disclosed	1,265	86.7	
OVCLHIV household has CHF card			<0.001
No	615	67.8	
Yes	1,365	93.1	
Number of household visits			<0.001
<20 visits	274	0.0	
20+ visits	1,706	98.9	
OVCLHIV sex			0.104
Female	1,016	86.5	
Male	964	83.9	
OVCLHIV age			0.017
00–04 years	323	80.2	
05–09 years	666	85.7	
10–14 years	991	86.6	
School enrolment status			0.017
In school	1,264	86.0	
Not school aged	244	78.7	
Out of school	205	85.4	
Unknown	267	87.6	
OVCLHIV adherence to ART in the last 6 months			<0.001
Not adherent	101	4.0	
Adherent	1,879	89.6	
Caregiver sex			0.572
Female	1,424	85.5	
Male	556	84.5	
Caregiver age			0.004
18–24 years	15	100.0	
25–45 years	886	83.4	
46–65 years	832	88.1	
66+ years	247	81.4	
Household food security			<0.001
Not secured	364	46.4	
Secured	1,616	94.0	
Household size			0.208
2 people	706	87.1	
3 people	490	83.3	
4 people	370	83.5	
5 people	414	86.0	
Place of residence			0.831
Rural	1,257	85.1	
Urban	723	85.5	

Results show that overall, 85.3% (*n* = 1,688) of the OVCLHIV studied were virally suppressed after 24 months. Viral suppression rate was higher among OVCLHIV attending PLHIV groups than those not attending the PLHIV groups (94.1 vs. 5.6%, *p* < 0.001). Similarly, viral suppression rate was higher among OVCLHIV whose HIV status was disclosed than in those whose HIV status was not disclosed (86.7 vs. 0.0%, *p* < 0.001). OVCLHIV with health insurance (iCHF) were significantly associated with higher virologic suppression compared to their uninsured counterparts (93.1 vs. 67.8%, *p* < 0.001). OVCLHIV who were visited by the CCW more frequently within the 24 months period were more likely to become virally suppressed compared to OVCLHIV who were visited less frequently within the same period (98.9 vs. 0.0%, *p* < 0.001) (Table 2).

The association between OVCLHIV viral suppression and demographic characteristics indicated that viral suppression was positively associated with OVCLHIV's age, ranging from 80.2% among the youngest OVCLHIV (aged 0–4 years) to 86.6% among those aged 10–14 years (*p* = 0.017). OVCLHIV with ART adherence level of >95% were more likely to be virally suppressed, compared to those who were less adherent to ART in the last 6 months (89.6 vs. 4.0%, *p* < 0.001) (Table 2).

OVCLHIV from food secure households were more likely to be virally suppressed, compared to those from insecure households (94.0 vs. 46.4%, *p* < 0.001). Other caregiver characteristics such as sex, household size and place of residence were not significantly associated with OVCLHIV virological suppression (Table 2).

### Results from the multivariable analysis

Table 3, presents multivariable logistic regression model of factors associated with viral load suppression among Tanzanian OVCLHIV on treatment. Adjusted odds ratios (ORs) and their corresponding 95% confidence intervals (CIs) and *p*-values are presented.

**Table 3:** Multivariable logistic regression analysis of factors associated with viral suppression at 24 months among OVCLHIV on ART in Tanzania (*N* = 1,980).

	**Adjusted odds ratio (aOR)**	**95% Confidence interval (CI)**		***P*-value**
		**Lower limit**	**Upper limit**	
**Attending PLHIV group**				
No	1.00	—	—	—
Yes	411.25	168.2	1,005.4	<0.001
**OVCLHIV household with iCHF card**				
No	1.00	—	—	—
Yes	6.05	3.28	11.15	<0.001
**OVCLHIV adherence to ART in the last 6 months**				
Not adherent	1.00	—	—	—
Adherent	148.96	42.6	520.6	<0.001
**OVCLHIV sex**				
Female	1.00	—	—	—
Male	1.20	0.73	1.96	0.473
**OVCLHIV age**				
00–04 years	1.00	—	—	—
05–09 years	0.26	0.05	1.25	0.092
10–14 years	0.22	0.05	1.07	0.061
**School enrolment status**				
In school	1.00	—	—	—
Not school aged	0.53	0.10	2.91	0.467
Out of school	0.60	0.28	1.29	0.190
Unknown	0.81	0.40	1.63	0.545
**Caregiver sex**				
Female	1.00	—	—	—
Male	0.86	0.50	1.49	0.586
**Household food security**				
Not secured	1.00	—	—	—
Secured	14.93	8.76	25.45	<0.001
**Household size**				
2 people	1.00	—	—	—
3 people	0.83	0.42	1.67	0.609
4 people	1.23	0.54	2.80	0.618
5 people	2.97	1.25	7.07	0.014
**Place of residence**				
Rural	1.00	—	—	—
Urban	0.90	0.53	1.53	0.699

Results show that after adjusting for other variables in the model, OVCLHIV attending PLHIV groups or age-appropriate clinic were more than 411 times more likely to achieve viral suppression than those not attending the groups (aOR = 411.25, 95% CI 168.2–1,005.4, *p* < 0.001). Similarly, OVCLHIV in households with health insurance were 6 times more likely to be virally suppressed than those who did not have health insurance (aOR = 6.05, 95% CI 3.28–11.15, *p* < 0.001). OVCLHIV with >95% adherence to ART were 149 times more likely to be virally suppressed than those not adherent to ART (aOR = 148.96, 95% CI 42.6–520.6, *p* < 0.001).

OVCLHIV living in food secure households were 15 times more likely to be virally suppressed than those in food insecure households (aOR = 14.93, 95% CI 8.72–25.45, *p* < 0.001). OVCLHIV in households with 5 members were almost thrice more likely to achieve viral suppression than those in smaller families of 2 people only (aOR = 2.97, 95% CI 1.25–7.07, *p* = 0.014).

OVCLHIV sex, age, and school enrollment status; caregiver sex, and age; and place of residence were not significantly associated with OVCLHIV's viral suppression in the multivariable analysis (Table 3).

## Discussion

This study assessed the determinants of viral load suppression among OVCLHIV aged 0–14 years who were on HIV treatment in Tanzania. The OVCLHIV were receiving HIV interventions services for 24 months, from October 2018 to September 2020. Findings showed that overall, 85.3% of the 1,980 OVCLHIV analyzed had achieved viral suppression at the end of the 24 months period. Although this rate was 4.7% lower than the UNAIDS' third 90 of viral load suppression target of 90% by 2020 (20), it was remarkably higher than 47% observed among children aged 0–14 years reported by the UNAIDS 2020 (2).

Another study in Tanzania among 0–24-year-olds found a viral load suppression rate of 65.8% (21), which is also lower than the rate seen in this study. Viral suppression rate in this study was comparable to the rate reported in Western Kenya (83%), although the study was based on the general population (22). One study in Rwanda reported a viral suppression rate of 61% among adolescents aged <19 years (23), and another study in Uganda observed a viral suppression rate of 63% (24). In both countries, the viral load suppression rates were lower than that observed by the current study.

The higher rate of viral load suppression observed in this study is attributable to attendance in PLHIV groups, health insurance, disclosure of HIV status at 8+ years and receiving household monitoring visits by CCWs. These interventions are designed to support retention and promote long-term ART adherence for ultimate achievement of viral load suppression. Therefore, given the benefits of the interventions, this study underscores the need for endless sustainability of the community-based interventions and inclusion of all OVCLHIV in HIV care and treatment services in order to increase the viral load suppression rate toward 100%.

Regarding factors influencing VLS, this study observed that OVCLHIV who received community-based interventions were more likely to be virally suppressed than those who did not.

Participation in OVCLHIV support groups was more likely to improve VLS than non-participation. This result is explained by the fact that OVCLHIV attended psychosocial activities, OVCLHIV and their caregiver received counseling sessions by trained provider, attended support-group sessions activities to discuss retention and adherence to ART. A study in South Africa found higher viral suppression rates among adolescents and young adults attending the adolescent clinic (91%) vs. adolescents attending the standard pediatric clinic (80%) (25). HIV viral suppression among patients aged 5–19 years attending sites with PLHIV clubs was higher (60%) compared to those attending health facilities with no PLHIV clubs (49%) (26).

In this study, viral suppression rates 85.3% (*n* = 1,688) resulted by CCWs close monitoring to OVCLHIV on retention and adherence to ART during household monthly visits. Thus, OVCLHIV rarely missed their appointment to the health facilities for routine reviews and intervention. Provision of OVCLHIV referral for HIV services (such as ART adherence, HIV disclosure support, opportunistic infections treatment) by CCWs also improved retention on ART. Monitoring OVCLHIV who receive ART is crucial to ensure successful treatment management, treatment of opportunistic infection and identify problems related to ART retention and adherence and determine change of ART regimens in case of treatment failure (7, 8, 17).

Taken together, the interventions favorably influenced retention, and adherence to ART among the OVCLHIV, and ultimately contributed to viral suppression. Missed clinic and ART refill appointments have negative implication on ART intake hence poor adherence to ART. Poor retention and adherence to ART are major factors which influence viral suppression. High viral suppression can largely be explained by ART retention and adherence to ART (21).

This study showed results of other factors which had significant predictive effect of OVCLHIV viral suppression. OVCLHIV from food secure households were more likely to be virally suppression than those from food insecure households. This finding is consistent with several studies all of which observed food insecurity as a barrier to ART adherence (27, 28). Since adherence is a prerequisite for viral suppression (21), improving adherence means contributing to viral suppression. Reasons for poor adherence to ART due to food insecurity were observed in Uganda and included that ART increases appetite for food; without food, ART's side effects exacerbate; and that there are competing demands between costs of food and medical expenses, among others (27). Therefore, there is a need to integrate nutritional support or food security intervention in HIV treatment efforts as an imperative dimension for improved treatment outcomes.

## Limitations

During the 24 months of service provision, some community volunteers had dropped out and were replaced by new community volunteers. Volunteer changes affected service delivery because caregivers take time to build trust and establish new volunteer- caregiver relationship for disclosure purpose. Since HIV disclosure status requires building trust overtime, it affected timely disclosure of OVC HIV status to enable service delivery. Volunteers change over time were due to reasons such as reallocation, marriage, death and drop out.

A minority of OVCLHIV 16.4% (48) had their next HVL test 3 months after the study duration (September 30th, 2020). Having OVCLHIV most updated VL results before the end of the study duration might have affected OVCLHIV viral load suppression results positively.

This study relied on self-reported ART adherence, which is subject to recall bias. OVCLHIV adherence to ART was self-reported by OVCLHIV or their caregiver by asking a recall question on whether OVCLHIV has missed her/his ART medication during the past 30 days. The limitation on self-reported question is the accuracy to recall the number doses/pills that were missed in the last 30 days which sometimes might not be accurate.

Food security data used for this study was obtained once by the program at 12 months during 24 months of the study duration. Therefore, data used for analysis in this study had no additional data to make comparison of OVCLHIV household's food security status over time in relation to viral load suppression.

## Conclusions

Although the rate of viral suppression was high, OVCLHIV who were reached by the different community-based interventions to promote retention and adherence (PLHIV support groups or age-appropriate clinics, health insurance, and adherence support through household visits by CCWs) had higher rates, and they were more likely to be virally suppressed than those who were not reached by the interventions. To advance viral suppression, efforts should be made to ensure that all OVCLHIV are reached by the interventions as well as integrating food support in HIV treatment interventions.

Community-based programs/interventions to promote retention and adherence should be prioritized as a more beneficial way to positively influence viral suppression. The government of Tanzania should prioritize delivering of tailor-made HIV service package to OVCLHIV with limited access to comprehensive care and treatment services. Scaling up of PLHIV support groups or age-appropriate clinics, health insurance coverage, and adherence support through household visits by CCWs will foster retention on and adherence to ART, reduce loss to follow up and increase OVCLHIV viral load suppression. The government should also integrate nutritional support or food security interventions in OVCLHIV treatment efforts as an imperative dimension for improved HIV treatment outcomes.

## Data availability statement

The raw data supporting the conclusions of this article will be made available by the authors, without undue reservation.

## Ethics statement

The studies involving human participants were reviewed and approved by National Institute for Medical Research (NIMR). Written informed consent to participate in this study was provided by the participants' legal guardian/next of kin.

## Author contributions

AA: problem conceptualization, study design, statistical analysis, drafting the manuscript, and critical review of the manuscript for intellectual content. AE and JC: statistical analysis, literature review, drafting the manuscript, critical review of the manuscript for intellectual content, and review of subsequent versions. AB, RF, AT, and LK: critical review of the manuscript for intellectual content. RM: data management and critical review of the manuscript for intellectual content. All authors contributed to the article and approved the submitted version.
